# Supplementary material for: Combination of Fetal Fraction Estimators Based on Fragment Lengths and Fragment Counts in Non-Invasive Prenatal Testing
Source: Int J Mol Sci. 2019 Aug 14;20(16):3959. doi: 10.3390/ijms20163959 (PMC6719007; doi:10.3390/ijms20163959)
Supplement: Supplementary file 1 [file ijms-20-03959-s001.pdf]

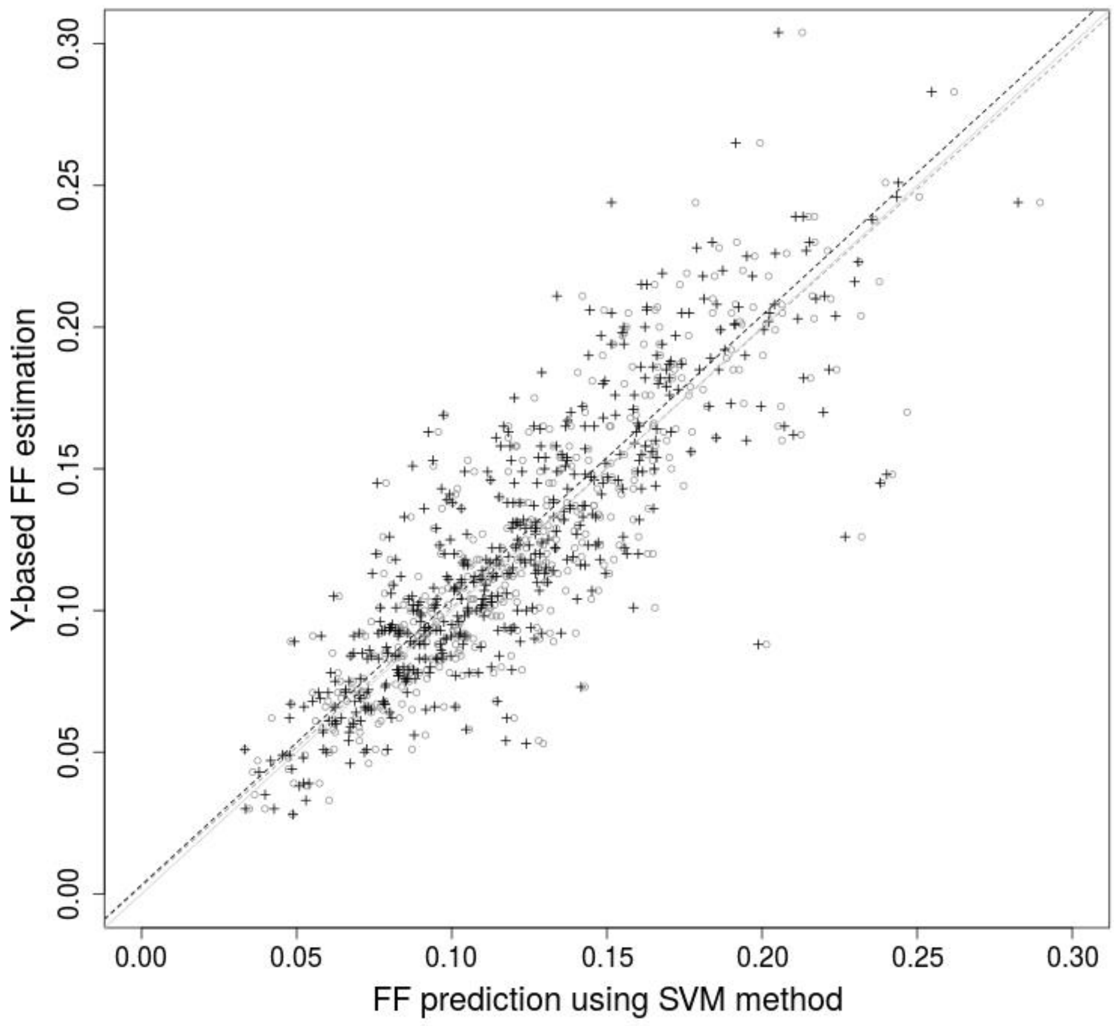

Supp figure 1: Linear dependency of Y-based FF estimation and FF prediction using SVM method. Grey circles denote testing dataset with standard training, in contrast to black crosses denoting testing dataset where training samples with FF < 10% were sampled (weighted) 2x. Dashed lines represent overall trend of the prediction (using of more samples with low fetal fraction causes underestimation of FF especially in samples with high FF).

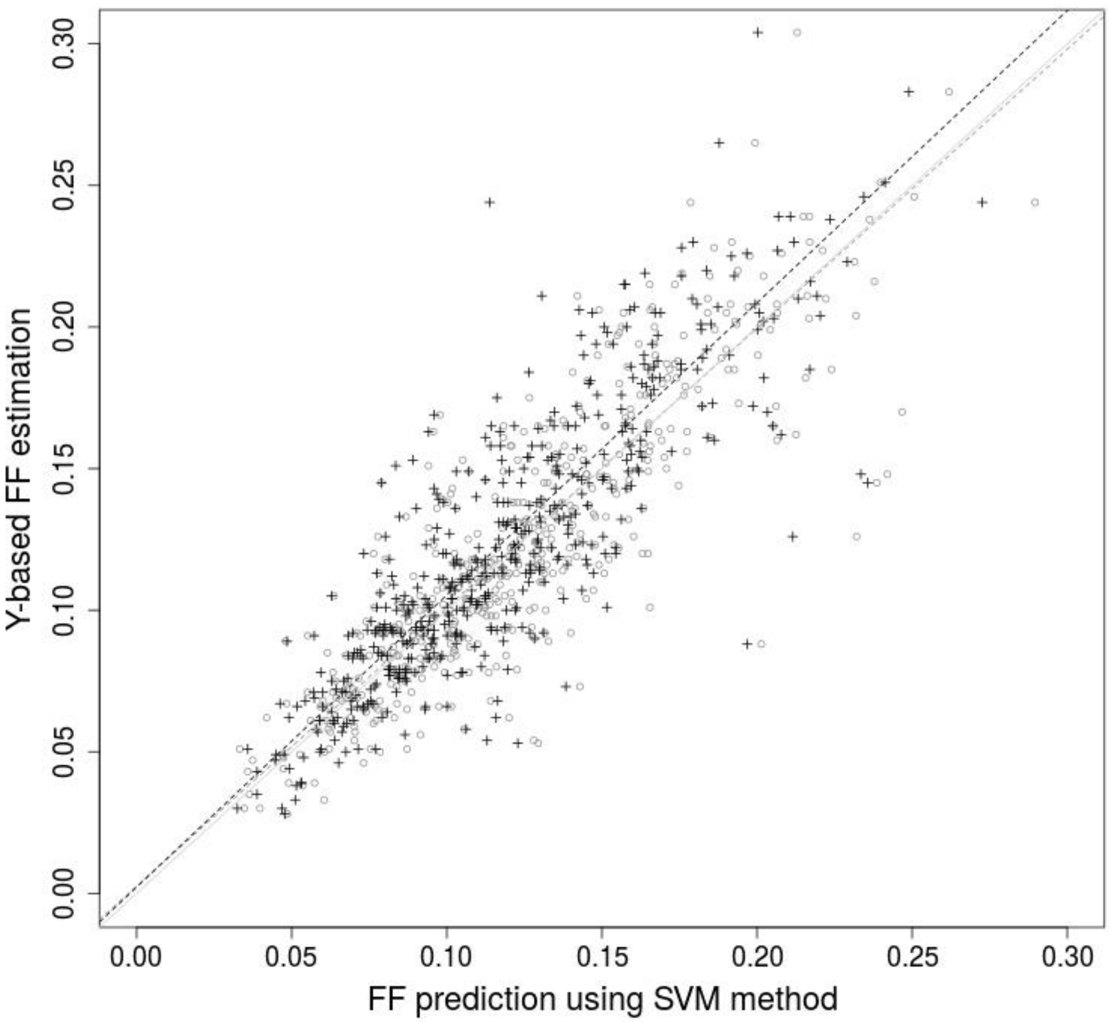

Supp figure 2: Linear dependency of Y-based FF estimation and FF prediction using SVM method. Grey circlets denote testing dataset with standard training, in contrast to black crosses denoting testing dataset where training samples with FF < 10% were sampled (weighted) 3x. Dashed lines represent overall trend of the prediction (using of more samples with low fetal fraction causes underestimation of FF especially in samples with high FF).

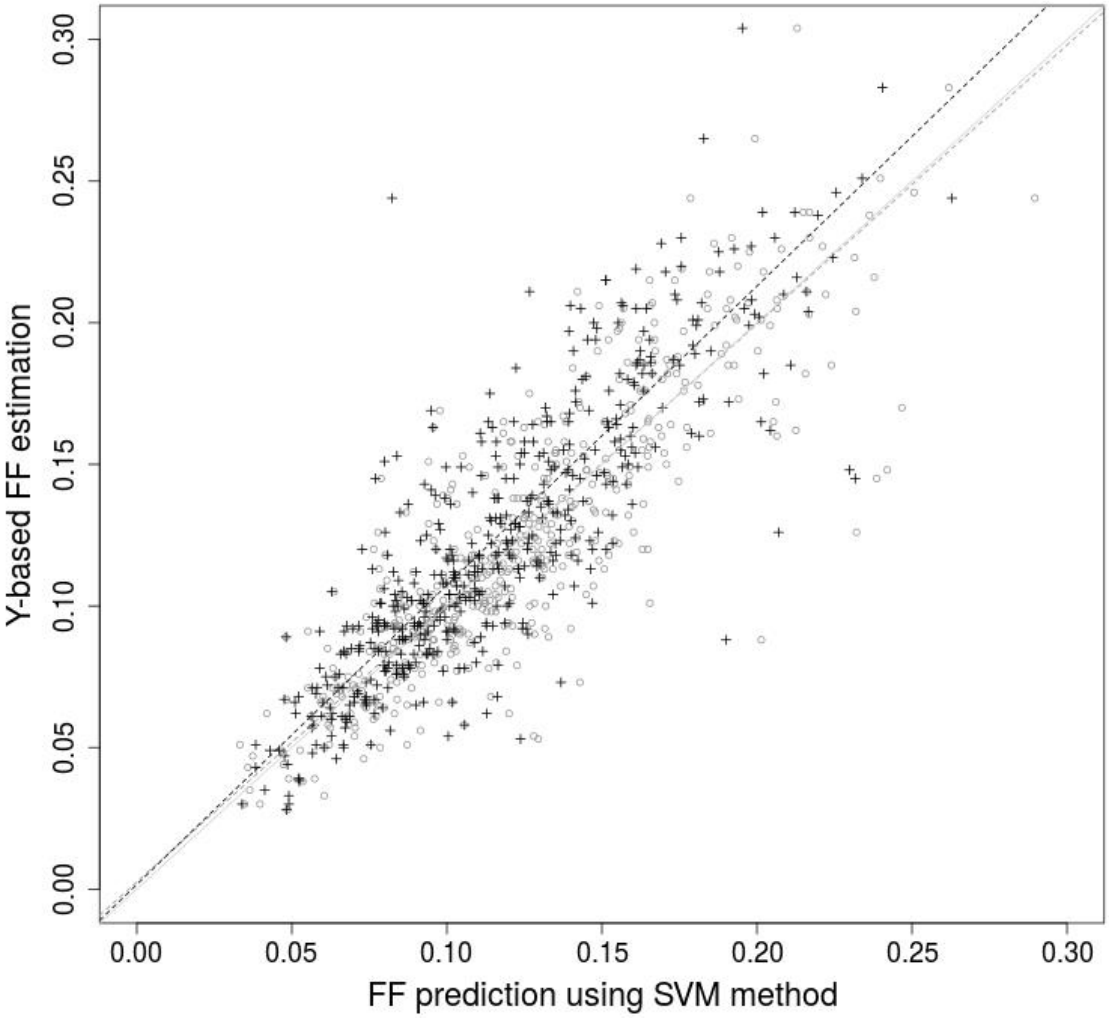

Supp figure 3: Linear dependency of Y-based FF estimation and FF prediction using SVM method. Grey circles denote testing dataset with standard training, in contrast to black crosses denoting testing dataset where training samples with FF < 10% were sampled (weighted) 4x. Dashed lines represent overall trend of the prediction (using of more samples with low fetal fraction causes underestimation of FF especially in samples with high FF).

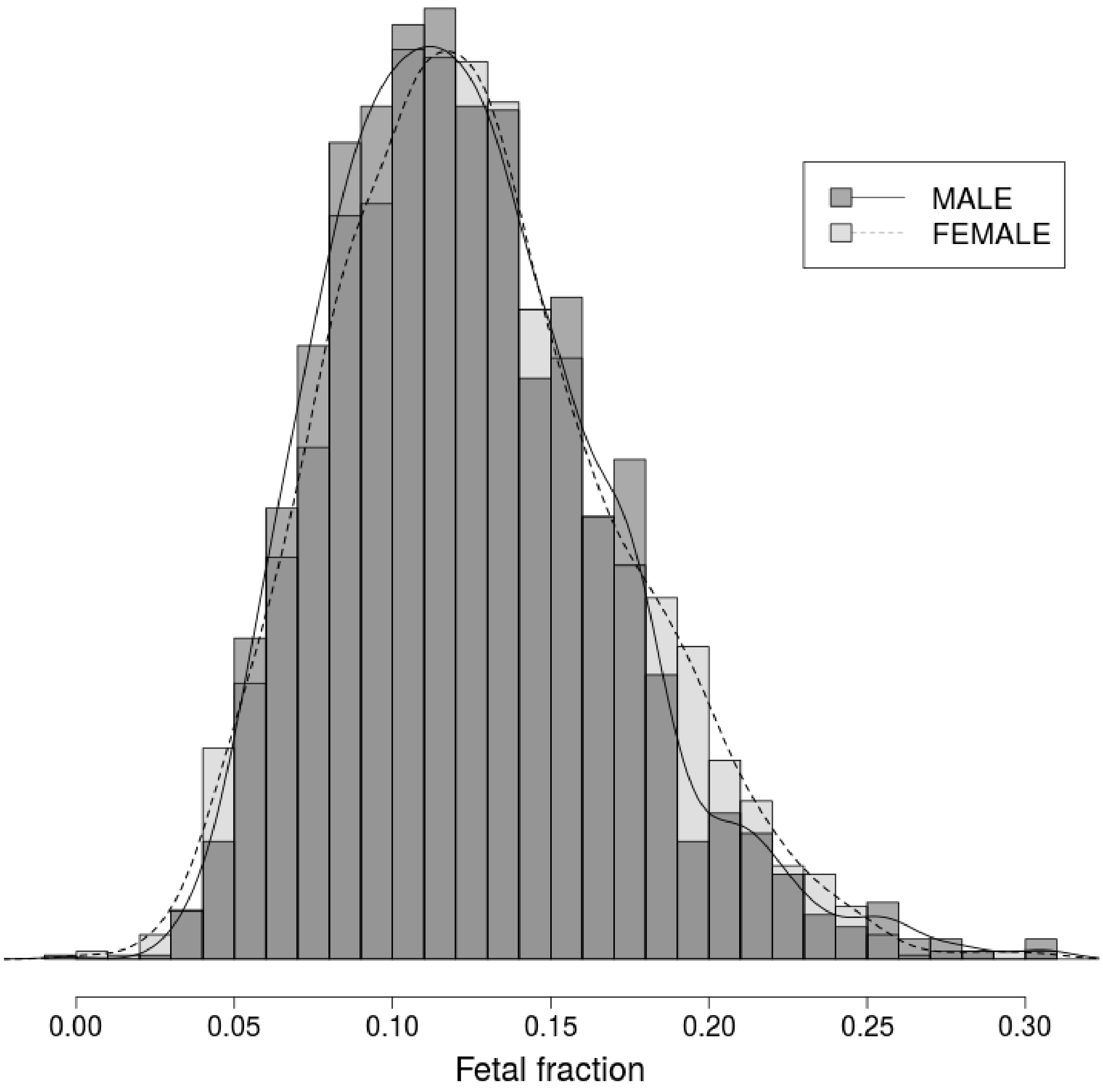

Supp figure 4: Histogram of male and female FFs using combined approach. Continuous line and dashed line represent density for male and female samples, respectively.

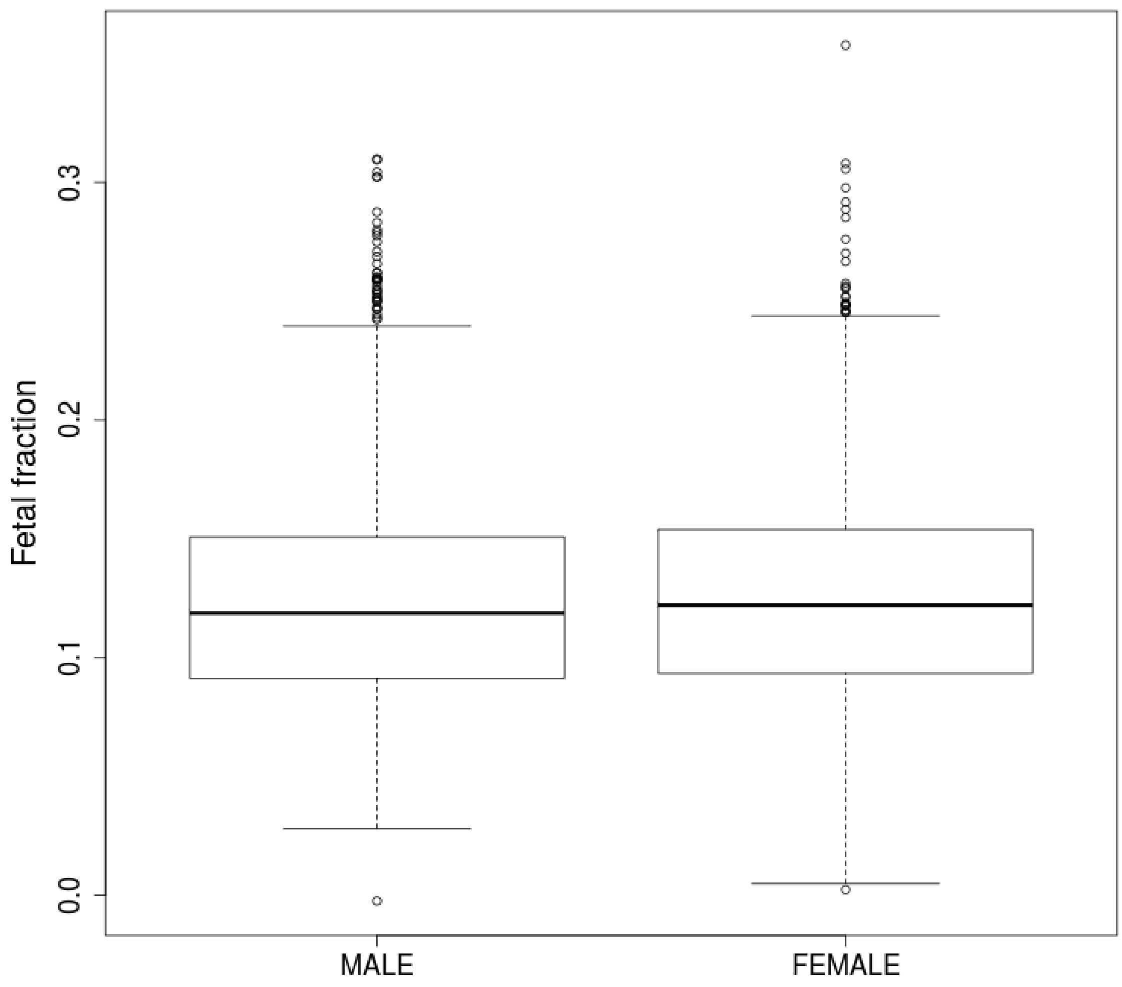

Supp figure 5: Boxplots of male and female FFs using combined approach.
